# Prevalence and correlates of burnout among physicians in a developing country facing multi-layered crises: a cross-sectional study

**DOI:** 10.1038/s41598-022-16095-5

**Published:** 2022-07-23

**Authors:** Dalal Youssef, Janet Youssef, Linda Abou-Abbas, Malak Kawtharani, Hamad Hassan

**Affiliations:** 1 Bordeaux Research Center for Population Health, Institut de santé publique, d’épidémiologie et de développement (ISPED), Bordeaux University, Bordeaux, France; 2 Preventive Medicine Department, Ministry of Public Health, Beirut, Lebanon; 3 Clinical Trial Program, Ministry of Public Health, Beirut, Lebanon; 4 Al Zahraa Hospital University Medical Center, Jnah, Lebanon; 5 Epidemiological Surveillance Unit, Ministry of Public Health, Beirut, Lebanon; 6 Neuroscience Research Center, Faculty of Medical Sciences, Lebanese University, Beirut, Lebanon; 7 Ministry of Public Health, Beirut, Lebanon

**Keywords:** Psychology, Diseases, Health care, Health occupations, Risk factors

## Abstract

Burnout among physicians is a serious concern that cultivates its seeds during their education. This study assessed the prevalence of burnout among Lebanese physicians and explored its correlates and the combined effects of the pandemic and the economic crisis on burnout. A web-based cross-sectional study was conducted in December 2020 using a snowball sampling technique. Moderate and high levels of burnout hit 90.7% of the physicians where personal, work-related, and client-related burnout were detected among 80.4%, 75.63%, and 69.6% of them respectively. A strong association was found between the higher level of burnout and female gender, younger age, being single, having a dependent child, living with an elderly or a family member with comorbidities, and insufficient sleeping hours. Physicians’ specialties, working in a public health facility, limited years of professional experience, lack of previous experience in a pandemic, and extensive working hours were also associated with increased burnout. Furthermore, low income, working in the frontline, higher threat perception, and fear of COVID-19 were contributing to higher burnout. The combined effect of threat perception and financial hardship significantly increased burnout levels. The alarming burnout level detected among physicians urges health authorities to take prompt actions to enhance the physicians’ well-being.

## Introduction

In recent years, burnout syndrome has been a major concern widely discussed in the area of occupational health^1,2^. It was described as a state of physical and emotional exhaustion resulting from extended exposure to a stressful and demanding situations work environment^3,4^. It may occur in a very wide range of work contexts, and in particular in demanding jobs such as healthcare providers^5–7^. Healthcare workers (HCWs) belong to the most devoted servants to humanity which gives them a lifetime of professional gratification^8,9^. However, healthcare was listed among the top high-stress professions that provoke a high level of burnout^5^. Despite the variation in the extent of burnout among HCWs, several studies found that all medical staff including physicians^10,11^, pharmacists^12^, nurses^13,14^, and lab technicians^15^ experienced burnout.

Of note, physicians are among the top potential candidates for burnout^16^. Notably, burnout among physicians begins to cultivate its seeds during their education period, goes along with the residency, and finally matures and crowned their practicing life^17–19^. This could be ensuing of the exposure of physicians to high levels of work distress, persistent tension, extensive working hours, a wide range of tasks, and interaction with patients and their relatives, and colleagues as well^20,21^. They also deal regularly with several complex situations, including responsibility for the health of patients, high patient and family expectations, patients’ and families’ aggressive behaviors complaints and high expectations, and coping with death and injury^22–24^. Physicians who encounter these issues are more likely to have psychological and physical exhaustion which leads them to be cynical about their work^25–27^.

Several studies conducted before the COVID-19 pandemic have indicated a high prevalence of burnout among physicians^28–30^. The prevalence of burnout among physicians varies between countries; ranging from 3.7 to 54.1%^29,31,32^. In Arab countries, such prevalence ranged from 12.6 to 41.94%^33^. Another study estimated that one in every three physicians would suffer from burnout at a given time^34,35^. Of note, burnout among physicians has devastating personal and professional consequences and could incite them towards turnover, early retirement, and poor job performance^36,37^. Besides, it impacted negatively the quality of care provided to patients and increased the risk for medical errors^38–40^.

In the era of the COVID-19 pandemic, the prevalence of burnout among physicians is snowballing. Physicians experienced ever-increasing pressure in their daily lives, particularly at their work^41–43^. This upsurge was reported in some studies conducted worldwide^44,45^. Similar to other countries, Lebanon experienced many challenges imposed by the COVID-19 pandemic on its healthcare system which was already in a fragile state even before the pandemic^46^, the economic collapse^47^, and the Beirut blasting^48^. It was overwhelmed by the humanitarian crisis revealed by the influx of more than one million Syrian refugees^49^. However, the COVID-19 pandemic overlapped with an economic crisis that has its roots in the aftermath of the civil war goaded by corruption and mishandling of the country’s resources^50^. This economic crisis was ranked by the World Bank among the world’s three worst crises since the mid-1800s affecting living standards where the Lebanese pound has lost more than 90% of its value since the fall of 2019^51^. This was later followed by the devastating Beirut blast, which was coupled with a meteoric soar in COVID-19 infections and hospitalizations where ICU occupancy in the hospitals touched 95% in January 2021^52^. In comparison with other HCWs, physicians bear the large toll of the pandemic^53^. In addition, the growing number of physicians diagnosed with COVID-19 unveiled gaps in policies and laws intended to warrant physician safety such as coverage for healthcare, disability, and death^54^. As a result of these consecutive and combined events, Lebanese physicians are leaving to find a better life elsewhere^55^. In such a typical context of multiple calamities that fueled mental health problems and burnout; it is of great interest to assess the level of physical and psychological burnout experienced by Lebanese physicians in its three domains: personal, work-related, and client-related burnout using a recognized free-of-charge validated tool and to understand as well its determinants in order to prevent such syndrome and reduce its negative impact. Of note, concerns about the pandemic^56^ and financial wellbeing^57^ were able both to instigate psychological illnesses and could interact and increase the burnout among physicians.

This study aimed to assess the prevalence of burnout among Lebanese physicians stranded amid the mixture of crises, along with how sociodemographic factors, work-related factors, economic factors, and pandemic-related factors affect the intensity of burnout. Besides, we targeted to assess the combined effects of the COVID-19 pandemic and economic crisis on burnout.

## Methods

### Study design and population

A web-based quantitative cross-sectional study was conducted among Lebanese physicians from the eight Lebanese provinces using a snowball sampling technique. It was conducted in December 2020. Participants were identified via professional groups and health facilities and were electronically invited to participate.

Physicians were contacted via phone call and notified about the survey and its purpose. Upon their agreement to participate, an online questionnaire using a Google form was sent to them via email or WhatsApp as per their preference. They were invited if possible to disseminate the link of the survey among their colleagues. All practicing Lebanese physicians who had access to the internet were eligible to be part of the study. Physicians who are not practicing currently, those who were out of the country at the time of the survey, retired physicians, interns, and those who refused to give informed consent were excluded.

### Sample size calculation

Using an estimated population of 10,918 physicians^58^, an expected response of 50%, a 95% confidence level, and an estimated absolute error of 5%, the requisite sample size was calculated using the RAOSOFT digital sample size calculator which yielded the least required sample size of 372 participants.

### Ethical consideration

The study has no foreseeable risks and written informed consent was obtained in an electronic format. The study was conducted following the standards issued by the World Medical Association’s Declaration of Helsinki guidance. The study was exempted from ethical approval by the Lebanese Ministry of Public Health. Participants were reassured that their participation is voluntary. All information was gathered anonymously and handled confidentially. The study design assured adequate protection of study participants and do not imply any risk for them. No reward was received by participants in return for participation^59^.

### Instrumentation

A questionnaire was developed in the Arabic and the English languages through Google forms. The utilized scales used were translated into Arabic following the guidelines concerning the forward and backward translation. A consensus was used to resolve inconsistencies between the original and translated versions. A pilot survey was also conducted on 15 physicians, and reformulations for some questions were made throughout its course. The answers to the pilot survey were excluded from the final data of this study. The finalized anonymous, self-administered questionnaire took 10 to 13 min to be completed and consisted of three sections: (a) basic sociodemographic characteristics, (b) work-related and exposure to COVID-19 variables, and (c) the measurements.

The first section collected sociodemographic data of the participants, including gender, age, marital status, specialty, urbanicity, health status, and living conditions. It also included questions about the history of illnesses and the health status of people living with the participant. Physicians were also asked about the type of health facility where they worked. The second section covered the topic of exposure to COVID-19 in addition to work-related variables. Physicians were queried to answer whether they have worked in the frontline, treated COVID-19 patients, and been tested or diagnosed as a COVID-19 case. They were also asked if they had a family member or a colleague infected by COVID-19 and had previous experience in a pandemic/infectious disease outbreak. Of note, the term “working in the frontline” referred to physicians who reported direct contact with suspected or documented COVID-19 infected patients while previous experience with pandemic/outbreaks referred to prior work of the physicians during infectious disease outbreaks such as SARS, MERS, H1N1, or Ebola^60^.

The third section consisted of four validated scales to objectively assess financial well-being, threat perception, fear of COVID-19 (FOC), and burnout. The scales were used after requesting permission from their copyright owners when required.

#### The perceived threat (TP) and altruistic acceptance of risk questionnaire

This tool was developed by Chong^61^ to assess threat perception among HCWs. It consisted of ten items where nine of these items described HCWs’ perception of COVID-19 threat and one item asked for altruistic acceptance of COVID-19 risk. Since this scale was previously used among Lebanese HCWs, thus it could be used to assess this aspect among Lebanese physicians^62^. Ratings were given based on a five-point Likert scale from one (strongly disagree) to five (strongly agree). Responses were dichotomized into positive responses ‘agree’ or ‘strongly agree’, while ‘strongly disagree’, ‘disagree’, and ‘not sure’ were considered negative. The Cronbach alpha of this scale in this study was equal to 0.703.

#### The FOC scale

This tool developed by Ahorsu consisted of seven items^63^ and scored on a five-point Likert scale from one (strongly disagree) to five (strongly agree). The score is calculated by summing the answers and ranges from 1 to 35. Higher scores indicated a large extent of fear of COVID-19. This scale was previously used to assess the fear of COVID-19 among the Lebanese population^64^. In this study, the Cronbach’s alpha for this scale was 0.769.

#### The InCharge financial distress/financial well-being scale (IFDFW)

This tool was developed by Prawitz^65^ including eight items measuring the perceived financial distress/financial well-being on a linear scale from one to ten. Higher scores reflect lower financial distress and higher well-being. Of note, the IFDFW scale was used before in Lebanon in a study assessing the mental health outcomes of the COVID-19 on the Lebanese population^64^. For this study, the reliability of this scale was checked^66^ and the Cronbach’s alpha for IFDFW was 0.85.

#### The Arabic version of the Copenhagen Burnout scale A-CBI

The validated Arabic version of the CBI which consisted of 19 items was used^67^. The CBI evaluates personal-related (PB) (six items), work-related (WB) (seven items), and client-related (six items) (CB) burnout. Of note, the term “clients” referred to patients in this study. Participants were asked to rate how often they felt exhausted. Ratings were given based on a five-point Likert scale. Each item was scored from 0 to 100 (0 = never, 25 = Seldom, 0 = Sometimes, 75 = Often, 100 = Always). Of note, some questions were answered using another five-point Likert scale (to a very high degree, to a high degree, somewhat, to a low degree, to a very low degree). The mean items score was calculated per scale. A cut-off of 50 was used to assess the prevalence of burnout among physicians. A score of more than 50 is considered a high or moderate burnout level whereas a score less than 50 signifies a low burnout level or its absence. The score was valid and reliable according to many previous studies^66^. In this study, the Cronbach’s alpha of this scale was equal to 0.879.

### Statistical analysis

The generated data through google forms were downloaded in an excel sheet, then transferred to SPSS® software (Statistical Package for Social Sciences) version 24.0 for analysis. No missing data were recorded since the response to all questions was mandatory. For descriptive analysis, frequency and percentage were used for categorical variables, and the mean and standard deviation for quantitative variables. The normality distribution of CBI items was confirmed by the calculation of skewness and kurtosis values. (< 1)^68^. For the bivariate analysis, to compare the means between the two groups, the Student’s T-test was used. Levene’s test was used to check the assumption of the homogeneity of variances before running a One-Way analysis of variance ANOVA to compare three groups or more.

To limit the possibility of getting a statistically significant test resulting from the run of many simultaneous independent and dependent statistical tests, post hoc analyses using Bonferroni correction were performed which sets the significance cut off at α/n. (α: error type 1 and n: number of tests)^69^. The correlation between continuous variables (burnout subscales and other scores used) was explored using the Spearman correlation coefficient and the gamma coefficient was calculated to explore the association between ordinal variables^70^. The Eta squared was used to compare means and the coefficient of variation (r-squared) to estimate the effect size of the correlations^71^.

The assumptions required before running the multivariable analysis were checked including the absence of multicollinearity, the residues normality, the homoscedasticity assumptions, and the linearity of the relationship. Then, four multiple linear regressions were performed using the stepwise method to identify the correlates of dependent variables (CBI subscales) in the whole sample and to reach the most parsimonious model. As for independent variables, all variables that showed a p-value < 0.2 in the bivariate analysis were introduced in the multivariable including sociodemographic, family, health, Fear of COVID-19, work-related and economics-related variables were also included. Based on the sample size, into account, the maximum number of variables allowed to be introduced in the analysis were taken into account. The R-squared and adjusted R-Squared were calculated for the full model, and the partial Eta squared for individual items. p-value < 0.05 was considered statistically significant.

To assess the interaction between the TP and the financial wellbeing (IFDFW) scales, a multivariate analysis using the General Linear Model was conducted on the same dependent variables using the enter method. The estimated marginal means were calculated for burnout among subjects according to their TP of COVID-19 and IFDFW (high/low categories). Of note, the dichotomization of the two variables (TP and IFDFW) into high and low categories was done according to the median of each scale.

### Informed consent

Informed consent for participating in the study was obtained digitally through Google Forms from all subjects, and all methods were carried out in accordance with the relevant guidelines and national regulations for the Non-clinical studies. Specifically, at the beginning of the questionnaire, participants were asked whether they agree to participate in the research in order to be included in the study. Participants were also informed that their participation was voluntary and that they had the right to leave at any time without providing any explanation. No incentives were provided to the study participants.

## Results

### Baseline information of the participants

**Figure1:**
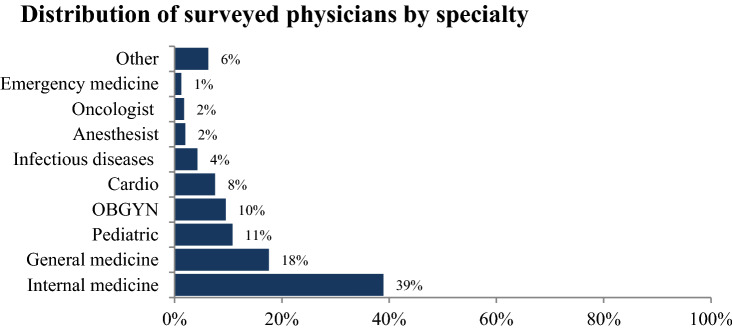
Distribution of surveyed physicians by specialty.

A total of 398 physicians participated in the survey. The majority of them were male (52.8%); married (60.1%), aged between 40 and 49 years old (43.2%), and residents of Mount Lebanon province (34.7%). Around half of participants had currently a dependent child (47.7%) or were living with the elderly (53%) or a family member with comorbidities at home (53.8%). More than two-thirds (69.85%) of surveyed physicians had a professional experience larger than 10 years and a previous experience in working in pandemics (74.12%). The highest percentage of participants were working on the frontlines (62.1%) and 51.9% of them were caring for COVID-19 cases. Only 15.3% of them had a previous history of COVID-19. However, 44.2% of the participants had a family member diagnosed with COVID-19 and 90.2% of them had a colleague diagnosed with COVID-19 (Table 1). Of note, the majority of surveyed physicians (39%) were specialized in internal medicine (Fig. 1).

**Table 1:** Socio-demographics characteristics of surveyed physicians (N = 398).

	n	%
**Gender**		
Male	210	52.80
Female	188	47.20
**Age (years)**		
Less than 40	143	35.90
40–49	172	43.20
≥ 50	83	20.81
**Marital status**		
Single	152	38.20
Married/engaged	239	60.10
Other (divorced or widowed)	7	1.80
**Residence**		
North & Akkar	66	16.60
Mount Lebanon	138	34.70
Beirut	105	26.40
South & Nabatyeh	45	11.30
Bekaa & Baalbeck-Hermel	42	11.00
**Working experience**		
Less than 10 years	120	30.15
10 years and more	278	69.85
**Previous experience in outbreak/pandemic/emergency**		
No	103	25.88
Yes	295	74.12
**Health facility type**		
Public	133	33.40
Private	265	66.60
**Presence of child at home**		
No	208	52.30
Yes	190	47.70
**Presence of elderly people at home**		
No	211	47.00
Yes	187	53.00
**Living with a family member with comorbidities**		
No	184	46.20
Yes	214	53.80
**Working on the frontline in the response to COVID-19**		
No	151	37.90
Yes	247	62.10
**Following up or caring for a COVID-19 case**		
No	191	48.10
Yes	207	51.90
**Personal history of COVID-19 diagnosis**		
No	337	84.70
Yes	61	15.30
**Family member/friend or colleague ever diagnosed with COVID-19**		
No	222	55.80
Yes	176	44.20
**Colleague ever diagnosed with COVID-19**		
No	39	9.80
Yes	359	90.20

*n* frequency, *%* percentage.

### Description of the scales

CBI had a mean of 65.34 (SD = 17.39) while the values for TP scale, FOC scale, and IFDFW were 35.53 (SD = 2.88), 17.88 (SD = 1.4), and 22.85 (SD = 7.64) respectively. The normality of the scales was assumed since skewness and kurtosis were lower than 1 and the sample size was larger than 300. The used scales showed good reliability; IFDFW (α = 0.85); FOC (α = 0.769); TP (α = 0.703) and CBI (α = 0.879). The lower scores of IFDFW reported in all items of the scale reflected higher financial distress and lower well-being. The highest burnout level was shown in WB (71.5 ± 16.33) followed by PB (64.8 ± 17.32) (Table 2).

**Table 2:** Descriptive statistics of the scales used in the study.

#	Scale items	Mean	S.D.
**IFDFW**	**Incharge financial distress/financial well-being scale (α = 0.85)**	22.85	7.64
IFDFW1	What do you feel is the level of your financial stress today?	2.98	1.48
IFDFW2	How satisfied you are with your present financial situation	2.78	1.26
IFDFW3	How do you feel about your current financial situation?	2.81	1.41
IFDFW4	How often do you worry about being able to meet normal monthly living expenses?	2.94	1.47
IFDFW5	How confident are you that you could find the money to pay for a financial emergency	3.16	1.58
IFDFW6	How often do you want to do something (eating outside, vacation, watching a movie, practicing a hobby….) and don’t go because you can’t afford to?	2.49	0.94
IFDFW7	How frequently do you find yourself just getting by financially and living paycheck to paycheck?	2.69	1.22
IFDFW8	How stressed do you feel about your personal finances in general?	3.00	1.48
**FOC**	**Fear of COVID-19 (α = 0.769)**	17.88	1.4
Fear1	I am most afraid of getting infected by COVID-19	3.82	0.40
Fear2	It makes me uncomfortable to think about Corona	2.03	0.33
Fear3	I am afraid of losing my life because of Corona	2.31	0.69
Fear4	When I watch news and stories about Corona on social media, I become nervous or anxious	3.69	0.49
Fear5	I cannot sleep because I’m worried about getting Corona	2.04	0.27
Fear6	My heart races or palpitates when I think about getting Corona	2.05	0.25
Fear7	My hands become clammy when I think about Corona	1.97	0.17
**TPS**	**Threat perception scale (α = 0.703)**	35.53	2.88
Threat1	My job puts me at great risk	4.02	0.63
Threat2	I feel more stress at work	4.00	0.47
Threat3	I have little control over whether I get infected or not	3.61	0.76
Threat4	I have little chance of survival if I were to get SARS	2.13	0.46
Threat5	I think of resigning because of SARS	2.17	0.45
Threat6	I am afraid I will pass SARS to others	3.93	0.40
Threat7	My family and friends are worried they get infected through me	4.07	0.32
Threat8	People avoid my family because of my work	3.83	0.98
Threat9	I am afraid of falling ill with SARS	4.04	0.50
ALtru1	I accept the risk of caring for SARS patient^**R**^	3.74	0.55
**CBI**	**Copenhagen Burnout Inventory scale (α = 0.879)**	65.34	17.39
	**Personal burnout (α = 0.921)**	64.80	17.32
PB1	How often do you feel tired?	63.57	17.87
PB2	How often you are physically exhausted?	63.94	17.84
PB3	How often you are emotionally exhausted?	65.01	17.72
PB4	How often do you think: ”I can’t take it anymore”?	65.45	15.47
PB5	How often do you feel worn out?	65.52	17.91
PB6	How often do you feel weak and susceptible to illness?	65.33	17.67
	**Work-related burnout (α = 0.832)**	71.50	16.33
WB1	Is your work emotionally exhausting?	72.49	16.36
WB2	Do you feel burnt out because of your work?	70.85	14.03
8WB3	Does your work frustrate you?	71.80	16.86
WB4	Do you feel worn out at the end of the working day?	71.83	16.29
WB5	Are you exhausted in the morning at the thought of another day at work?	71.04	15.32
WB6	Do you feel that every working hour is tiring for you?	71.55	14.49
WB7	Do you have enough energy for family and friends during leisure time? ^**R**^	70.98	15.76
	**Client burnout (α = 0.874)**	58.70	16.14
CB1	Do you find it hard to work with clients?	56.91	23.33
CB2	Do you find it frustrating to work with clients?	57.22	24.00
CB3	Does it drain your energy to work with clients?	55.65	19.18
CB4	Do you feel that you give more than you get back when you work with clients?	56.09	22.42
CB5	Are you tired of working with clients?	71.23	20.58
CB6	Do you sometimes wonder how long you will be able to continue working with clients?	55.09	19.30

*M* mean, *SD* standard deviation, *R* reversed coding.

### Prevalence of burnout among Lebanese physicians

**Figure 2:**
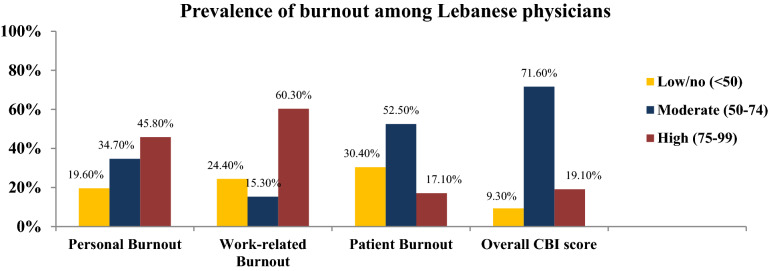
Prevalence of burnout and its three dimensions among Lebanese physicians.

Moderate and high level of burnout was detected among 90.1% of surveyed physicians, while 19.1% had a high level of burnout. PB ranked first among other burnout aspects (80.5%) with 45.8% of physicians reporting high PB levels. As for WB, it was detected in moderate and high levels among more than three-quarters of physicians (75.6%), where 60.3% exhibited a high level of WB. Moderate and high CB was found among 69.6% of participants (Fig. 2).

### Socio-demographic characteristics and burnout

Female gender, younger age, being single or divorced, physicians who had a dependent child, and those who live with elderly and family members with comorbidities had a significantly higher level of burnout. Similarly, all these sociodemographic variables were significantly associated with a high level of PB, WB, and CB except the age which was not significantly associated with the work-burnout dimension. The largest effect size was observed in age, marital status, and presence of a dependent child at home (Table 3).

**Table 3:** Association between socio-demographic characteristics and CBI subscales (N = 398).

		n (%), N = 398	Overall CBI			Personal burnout			Work-related burnout			Client-related burnout		
			Mean (SD)	p-value	Eta Squared	Mean (SD)	p-value	Eta squared	Mean (SD)	p-value	Eta squared	Mean (SD)	p-value	Eta squared
**Gender**				0.035	0.018		0.039	0.011		0.048	0.005		0.017	0.025
Male		210 (52.8%)	64.01 (10.96)			63.82 (16.55)			70.86 (15.33)			56.82 (15.47)		
Female		188 (47.2%)	65.64 (9.37)			65.78 (14.54)			72.21 (13.28)			60.37 (18.08)		
**Age (years)**				0.048	0.12		0.022	0.09		0.189	0.001		0.032	0.028
Less than 40		143 (35.9%)	66.56 (11.39	Ref		67.37 (15.66)	Ref		72.16 (15.79)			59.23 (19.38)		
≥ 40		255 (64.1%)	64.04 (9.71)	0.032		62.99 (15.84)	0.007		71.25 (13.29)			56.67 (16.38)		
**Marital status**				0.001	0.139		0.025	0.116		0.045	0.076		0.013	0.031
Single/divorced		159 (39.9%)	66.83 (10.72)			66.52 (14.84)			72.38 (15.22)			61.81 (15.16)		
Married/engaged		239 (60.1%)	63.12 (9.01)			61.31 (16.79)			68.87 (15.11)			59.65 (17.24)		
**Residence**				0.581	0.003		0.635	0.004		0.377	0.005		0.201	0.009
North/Akkar	66 (16.6%)		66.01 (10.06)			66.01 (12.08)			71.11 (14.97)			59.47 (17.89)		
Mount Lebanon	138 (34.7%)		64.23 (10.38)			67.23 (16.53)			70.62 (13.89)			53.78 (17.76)		
Beirut	105 (26.4%)		65.63 (11.05)			64.37 (16.66)			71.89 (15.11)			59.61 (18.05)		
South/Nabatyeh	45 (11.3%)		64.18 (7.61)			65.93 (13.91)			69.21 (12.77)			56.57 (13.04)		
Great Bekaa	42 (11%)		64.98 (8.01)			63.63 (12.25)			72.48 (11.67)			57.57 (12.39)		
**Presence of dependent children at home**				0.047	0.01		0.013	0.121		0.022	0.012		0.034	0.02
No		208 (52.3%)	64.38 (10.23)			62.62 (15.47)			70.75 (15.03)	Ref		57.05 (17.19)		
Yes		190 (47.7%)	66.40 (10.18)			67.19 (15.46)			72.18 (13.79)			60.5 (16.58)		
**Presence of elderly at home**				0.014	0.011		0.031	0.014		0.047	0.009		0.044	0.012
No		211 (47%)	63.18 (9.74)			63.62 (14.47)			70.75 (15.03)	Ref		58.32 (16.9)		
Yes		187 (53%)	68.61 (10.98)			67.89 (13.46)			72.18 (13.79)			60.1 (15.58)		
**Family member with comorbidities**				0.045	0.018		0.042	0.01		0.038	0.01		0.001	0.022
No		184 (46.2%)	63.98 (10.23)			63.62 (15.08)			70.75 (15.03)	Ref		56.05 (16.32)		
Yes		214 (53.8%)	67.10 (11.03)			66.84 (14.86)			72.18 (13.79)			60.5 (16.58)		

*N* frequency, *%* percentage, *SD* standard deviation, *Eta sq.* Eta squared, the mean was unstandardized, Great Bekaa included Bekaa and BaalbeckHermel province.

### Economic characteristics and burnout

Surveyed physicians who have private health coverage and those who subjectively classified themselves as having a low socioeconomic status currently had significantly higher burnout in all its aspects (PB, WB, and CB). Besides, physicians who earned less than two Million Lebanese pounds per month and those who considered that pandemic or economic crisis highly impacted their monthly income showed also high burnout. However, financial well-being (FWB) was negatively correlated with high burnout. Regarding burnout, the largest effect size was seen in low economic status after the pandemic and economic crisis, major impact of the economic crisis on the income and FWB (Table 4).

**Table 4:** Association between economic factors and CBI subscales (N = 398).

	n (%), N = 398	Overall CBI			Personal burnout			Work-related burnout			Client-related burnout		
		Mean (SD)	p-value	Eta sq.	Mean (SD)	p-value	Eta sq.	Mean (SD)	p-value	Eta sq.	Mean (SD)	p-value	Eta sq.
Socio-economic status after COVID-19/economic crisis*			< 0.001	0.149		< 0.001	0.121		< 0.001	0.152		0.029	0.132
Rich	3 (0.7%)	59.19 (11.23)	Ref		58.25 (15.33)	Ref		67.31 (16.75)	Ref		57.01 (15.76)	Ref	
Middle	125 (31.4%)	64.12 (9.75)	0.008		64.67 (16.76)			71.83 (18.15)			58.22 (16.32)	0.139	
Middle to low	273 (43.5%)	72.71 (10.34)	< 0.001		71.48 (17.32)			75.36 (15.23)			60.87 (16.03)	0.006	
**Current income**			< 0.001	0.046		0.035	0.081		0.006	0.076		< 0.001	0.064
< 2 million L.L	68 (17.1%)	67.87 (12.05)	Ref		66.44 (17.22)	Ref		73.678 (16.5)	Ref		64.04 (19.17)	Ref	
2–4 million L.L	172 (44.2%)	65.49 (11.4)	0.087		63.39 (14.85)	0.046		73.37 (13.45)	0.543		56.37 (20.26)	0.021	
> 4 million L.L	154 (38.7%)	63.07 (7.09)	< 0.001		61.98 (15.82)	0.021		68.91 (11.54)	0.001		54.92 (16.55)	< 0.001	
**Pandemic impact on income**			0.046	0.082		0.033	0.082		0.043	0.051		< 0.001	0.036
Minor	60 (15.1%)	63.11 (9.67)	Ref		62.92 (16.06)	Ref		68.41 (14.25)	Ref		56.96 (16.32)	Ref	
Moderate	199 (50%)	64.85 (9.68)	0.154		65.71 (16.01)	0.048		72.44 (14.67)	0.049		58.12 (14.97)	0.069	
Major	139 (34.9%)	68.24 (10.83)	0.009		66.94 (12.4)	0.031		74.50 (13.99)	0.018		60.53 (18.17)	< 0.001	
**Economic crisis impact on your income**			< 0.001	0.132		0.038	0.026		0.023	0.032		0.034	0.018
Minor	3 (0.7%)	58.47 (10.06)	Ref		61.13 (15.83)	Ref		69.25 (14.76)	Ref		56.98 (14.76)	Ref	
Moderate	57 (14.3%)	64.72 (11.28)	< 0.001		64.67 (16.04)	0.256		70.05 (13.18)	0.276		58.62 (15.23)		
Major	338 (84.9%)	73.01 (10.81)	< 0.001		68.03 (16.45)	0.009		75.12 (14.21)	0.003		60.31 (15.76)		
**Health coverage**			0.044	0.018		0.362	0.002		0.168	0.000		0.412	0.000
Public	23 (5.7%)	64.81 (9.41)			64.38 (15.51)			70.62 (14.11)			58.44 (15.45)		
Private (insurance, syndicates...)	375 (94.3%)	67.11 (12.03)			65.66 (16.07)			74.26 (14.72)			60.22 (20.34)		

Scale	Mean (SD)	Correlation (r)	p-value		Correlation (r)	p-value		Correlation (r)	p-value		Correlation (r)	p-value	
IFDWF scale	2.86 (1.43)	− 0.23	p < 0.01		− 0.278	< 0.01		− 0.212	< 0.01		− 0.17	< 0.05	

*N* frequency, *%* percentage, *SD* standard deviation, *Eta sq.* Eta squared, the mean was unstandardized.

### Occupational factors and burnout

Physicians working in public hospitals, those with limited professional experience (less than 10 years), and those who lacked a previous experience during pandemics had significantly higher levels of burnout compared to their counterparts. Furthermore, insufficient sleeping hours, extensive working hours, and physicians’ higher perception of COVID-19 impact on their work increased the overall burnout among participants. These factors had a large effect size related to the overall burnout. Similar occupational factors were associated with a high level of BP except for extensive working hours. In addition to the identified professional factors increasing burnout among physicians, working in hospitals located in urban areas had higher WB. In terms of CB, a higher level was associated with health facility type, previous pandemic experience, and extensive working hours (Table 5).

**Table 5:** Work characteristics and CBI subscales (N = 398).

	N (%) N = 398	Overall CBI			Personal burnout			Work-related burnout			Client-related burnout		
		Mean (SD)	p-value	Eta sq.	Mean (SD)	p-value	Eta sq.	Mean (SD)	p-value	Eta sq.	Mean (SD)	p-value	Eta sq.
**Health facility type**						0.035	0.011		0.012	0.029		0.03	0.006
Private	265 (66.6%)	62.5 (10.36)			62.98 (12.68)			69.11 (13.34)			56.64 (16.25)		
Public	133 (33.4%)	68.1 (10.06)			65.03 (11.76)			73.46 (14.22)			60.34 (16.8)		
**Location of the hospital**			0.143	0.001		0.511	0.002		0.018	0.009		0.308	0.000
Rural	109 (27.4%)	64.31 (11.22)			63.54 (14.71)			69.08 (15.18)			58.54 (17.05)		
Urban	289 (72.6%)	66.52 (10.83)			65.21 (15.12)			73.8 (14.27)			58.82 (16.94)		
**Working experience**			0.003	0.010		0.043	0.019		0.028	0.017		0.64	0.001
Less than 10 years	120 (30.1%)	68.25 (11.83)			67.99 (15.13)			73.15 (14.22)					
10 years and more	278 (69.8%)	62.64 (11.47)			63.02 (14.73)			68.13 (13.89)			58.48 (16.44)		
**Previous experience in outbreak/pandemic/emergency**			0.048	0.009		0.043	0.018		0.031	0.008		0.038	0.004
No	103 (25.8%)	65.81 (10.46)			65.39 (15.83)			73.59 (13.25)			59.25 (17.29)		
Yes	295 (74.1%)	62.74 (8.52)			61.54 (14.02)			67.64 (11.68)			55.66 (14.82)		
**Sleeping hours**			< 0.001	0.022		0.002	0.031		0.018	0.000		0.339	0.002
Less than 6 h	210 (57.7%)	69.03 (10.35)			67.53 (15.38)			67.48 (12.27)			57.88 (17.11)		
More than 6 h	168 (42.2%)	61.18 (11.22)			62.02 (13.17)			74.01 (14.31)			58.98 (16.89)		
**Extensive working hours**			0.011	0.017		0.876	0.000		0.022	0.012		0.43	0.019
No	99 (24.8%)	62.56 (9.08)			64.54 (13.51)			70.01 (14.34)			57.03 (16.25)		
Yes	299 (72.3%)	66.53 (10.86)			64.34 (14.72)			72.18 (15.12)			60.12 (16.8)		
**Economic crisis impact on your work**			< 0.001	0.053		0.038	0.026		0.023	0.032		0.234	0.001
Minor	13 (0.7%)	58.47 (10.06)	Ref		61.13 (15.83)	Ref		69.25 (14.76)	Ref		56.98 (14.76)		
Moderate	97 (14.3%)	64.72 (11.28)	< 0.001		64.67 (16.04)	0.256		70.05 (13.18)	0.276		58.62 (15.23)		
Major	288 (84.9%)	73.01 (10.81)	< 0.001		68.03 (16.45)	0.009		75.12 (14.21)	0.003		60.31 (15.76)		

*N* frequency, *%* Percentage, *SD* standard deviation, *Eta sq.* Eta squared, the mean was unstandardized.

#### Exposure, perception of COVID-19 threat, fear of COVID-19, altruism, health characteristics, and burnout

Having a good health status, a history of COVID-19 infection and altruism were significantly associated with a lower level of burnout in all aspects. FOC and higher TP were correlated with higher burnout among physicians. Similarly, participants who perceived a major impact of the pandemic on their daily life and their familial relationship reported higher levels of burnout. The largest effect size was found for the TP of COVID-19, altruistic and COVID-19 impact on familial relationships. Altruism was significantly associated with a decreased burnout in all its aspects (Table 6).

**Table 6:** Association between COVID-19 exposure, health characteristics, COVID-19 impact, and CBI subscales (N = 398).

	n (%), N = 398	Overall CBI			Personal burnout			Work-related burnout			Client-related burnout		
		Mean (SD)	p-value	Eta sq.	Mean (SD)	p-value	Eta sq.	Mean (SD)	p-value	Eta sq.	Mean (SD)	p-value	Eta sq.
**Health status**			0.002	0.021		0.035	0.011		0.012	0.029		0.043	0.008
Fair and Below	70 (17.6%)	68.1 (10.36)			66.28 (14.68)			74.16 (13.88)			59.34 (16.25)		
Good and above	328 (82.4%)	62.5 (10.67)			63.11 (14.76)			68.31 (12.94)			57.64 (16.8)		
**Working in frontline**			0.038	0.016		0.04	0.011		0.032	0.009		0.003	0.056
No	151 (37.9%)	63.5 (10.36)			62.9 (15.48)			69.41 (14.63)			56.64 (17.25)		
Yes	247 (61.1%)	67.1 (10.06)			66.28 (15.76)			73.16 (14.22)			60.34 (16.8)		
**Following up or caring for a COVID-19 case**			0.325	0.001		0.421	0.003		0.018	0.022		0.308	0.001
No	191 (48.1%)	64.17 (10.39)			65.54 (14.71)			70.08 (15.18)			57.54 (17.05)		
Yes	207 (51.9%)	66.32 (10.14)			64.21 (16.29)			72.8 (13.77)			59.82 (16.94)		
**Tested for COVID-19**			0.794	0.000		0.053	0.009		0.098	0.007		0.604	0.001
No	91 (22.9%)	65.64 (10.47)			63.97 (15.63)			69.31 (14.83)			58.48 (16.44)		
Yes	307 (77.1%)	65.25 (10.18)			67.58 (15.13)			72.15 (14.22)			59.43 (18.71)		
**History of COVID-19 diagnosis**			0.031	0.012		0.043	0.018		0.231	0.002		0.038	0.008
No	337 (84.7%)	65.81 (10.46)			65.39 (15.83)			71.79 (14.65)			59.25 (17.29)		
Yes	61 (15.3%)	62.74 (8.52)			61.54 (14.02)			69.84 (12.88)			55.66 (14.82)		
**A family member diagnosed with COVID-19**			0.549	0.001		0.762	0.000		0.989	0.000		0.394	0.002
No	222 (55.8%)	64.99 (10.49)			64.53 (15.58)			71.48 (14.54)			57.88 (17.11)		
Yes	176 (44.2%)	65.62 (10.05)			65.02 (15.67)			71.51 (14.31)			59.34 (16.87)		
**Colleague ever diagnosed with COVID-19**			0.245	0.004		0.39	0.003		0.004	0.048		0.293	0.002
No	39 (9.8%)	63.56 (8.56)			64.33 (15.44)			65.29 (14.33)			55.98 (14.61)		
Yes	359 (90.2%)	65.53 (10.41)			69.12 (16.68)			71.17 (14.26)			58.99 (17.21)		
**Pandemic impact on daily life**			0.021	0.013		0.038	0.026		0.009	0.028		0.042	0.018
Minor	42 (10.5%)	63.992 (8.76)	Ref		63.29 (15.42)	Ref		66.42 (15.49)	Ref		56.59 (13.92)	Ref	
Moderate	96 (24.2%)	64.17 (9.38)	0.213		65.27 (15.48)	0.079		68.72 (13.46)	0.213		59.37 (18.21)	0.038	
Major	260 (65.3%)	66.96 (10.86)	0.006		66.98 (16.10)	0.002		73.06 (14.54)	< 0.001		63.61 (15.71)	< 0.001	
**Pandemic impact on social relationships**			0.176	0.002		0.003	0.031		0.321	0.000		0.415	0.001
Minor	31 (7.8%)	64.17 (9.38)			61.33 (15.76)			70.23 (12.67)			57.33 (17.05)		
Moderate	185 (46.5%)	65.32 (9.56)			65.78 (15.02)			71.97 (14.13)			58.71 (16.94)		
Major	182 (45.7%)	66.23 (10.15)			69.45 (14.98)			72.33 (14.46)			59.82 (16.13)		
**Pandemic impact on family relationship**			0.115	0.025		0.412	0.003		0.298	0.002		0.765	0.000
Minor	71 (17.8%)	62.43 (8.89)	Ref		64.33 (15.76)			69.98 (12.67)			58.33 (15.05)		
Moderate	136 (34.2%)	65.62 (10.23)			64.78 (15.02)			71.19 (13.58)			58.71 (16.72)		
Major	191 (48%)	69.33 (10.86)			66.45 (14.98)			73.01 (14.36)			60.02 (15.89)		
**Altruistic: accepting the risk of caring for COVID-19 case**			0.030	0.123		0.044	0.015		0.005	0.042		0.018	0.010
No	77 (19.3%)	66.58 (10.61)			67.47 (16.59)			72.48 (14.18)			61.86 (17.85)		
Yes	321 (80.7%)	63.58 (8.56)			64.16 (15.29)			67.39 (14.67)			55.57 (12.71)		

Scales	Mean (SD)	Correlation (r)	p-value		Correlation (r)	p-value		Correlation (r)	p-value		Correlation (r)	p-value	
Fear of COVID-19	2.55 (0.34)	0.141	p < 0.01		0.203	p < 0.01		0.285	p < 0.01		0.364	p < 0.01	
Threat perception	3.53 (0.94)	0.326	p < 0.01		0.319	p < 0.01		0.138	p < 0.01		0.132	p < 0.01	

*N* frequency, *%* percentage, *SD* standard deviation, *Eta sq*. Eta squared, the mean was unstandardized.

#### Correlates of burnout and its subscales: a multivariable analysis

Higher overall burnout was associated with female gender, younger age, physician specialty, working in public hospitals, higher TP, insufficient sleeping hours, low income, extensive working hours, having a dependent child or family member with comorbidities, and limited professional experience. However, being married, financial well-being, good health, history of COVID-19, altruism, and previous pandemic experience were significantly associated with lower burnout. The full model could explain 76.1% of the overall burnout. PB was associated with younger age, female gender, having a single or divorced marital status, presence of an elderly, child at home, or family member with comorbidities. Higher TP, FOC, sleeping disturbance, extensive working hours, and low income were associated with higher PB. However, financial well-being, altruism, and good health were associated with lower PB levels. The full model could explain 67.2% of the PB. As for WB, similar factors were found positively associated with higher burnout along with the hospital’s type. The full model could explain 58.4% of the WB. In terms of CB, it was found that younger age, higher perception of threat, FOC, and low income were associated with higher CB. Similar to other aspects, altruistic and large professional experience and financial wellbeing were associated with a decreased level of CB (Table 7).

**Table 7:** Multivariable analyses: Correlates of CBI and its subscales.

Model	Standardized Coefficients Beta	p-value	Confidence interval		Adjusted R squared
			Lower bound	Upper bound	
**Correlates of the overall CBI**					0.761
Female gender	0.202	0.022	0.062	1.038	
Age (≥ 40 vs < 40 years)	− 0.167	0.001	− 2.377	− 0.132	
Marital status (married versus single/divorced)	− 0.496	0.010	− 2.466	− 0.332	
Specialty (other specialties vs ID/internal medicine)	− 0.876	0.048	− 2.321	− 0.514	
Hospital type (private vs public)	− 0.130	< 0.001	− 3.272	− 1.091	
Threat perception scale	0.478	0.001	0.187	0.742	
IFDFW scale	− 0.222	0.044	− 1.934	− 0.048	
Sleeping hours (≤ 6 h vs > 6 h)	0.169	0.038	0.091	0.563	
Low income	0.318	< 0.001	1.920	2.204	
Health status (good vs poor)	− 0.123	0.029	− 1.642	− 0.052	
Child at home (yes vs no)	0.397	0.018	0.139	1.121	
Family member with chronic disease (yes vs no)	0.104	0.665	0.762	1.195	
Working in the frontline (yes vs no)	0.318	0.004	0.757	1.089	
Diagnosed as COVID-19 case (yes vs no)	− 0.185	0.042	0.582	0.101	
Previous experience of working in outbreaks (yes vs no)	− 0.289	< 0.001	− 0.934	− 0.048	
Work experience (small vs large)	0.092	0.560	0.026	1.567	
Fear of COVID-19	0.311	< 0.001	0.431	0.912	
Altruistic (yes vs no)	− 0.167	0.006	− 0.476	− 0.087	
Extensive working hours	0.131	< 0.001	0.182	0.626	
Sleeping hours (less than 6 h vs more than 6 h)	0.299	0.018	0.171	0.533	
**Correlates of the personal burnout**					0.672
Age (> 40 years)	− 0.110	0.048	− 5.272	− 0.091	
Marital status (single/divorced vs married)	0.222	0.022	0.839	− 0.162	
Gender (female)	0.478	0.001	0.187	0.742	
Health condition (good vs bad)	− 0.167	0.001	− 2.377	− 0.132	
Presence of child at home (yes vs no)	0.496	0.010	0.332	0.866	
Family member with comorbidities (yes vs no)	0.318	< 0.001	1.920	2.204	
Presence of elderly at home (yes vs no)	0.297	0.018	0.139	1.121	
Threat perception scale	0.215	0.046	0.186	1.267	
Altruistic	− 0.011	0.016	0.762	1.195	
Extensive working hours	0.218	0.004	0.757	1.089	
Low income	0.779	< 0.001	0.101	0.582	
Fear of COVID-19	0.540	0.036	0.230	1.260	
IFDFW scale	− 0.345	< 0.001	− 1.340	− 0.138	
Sleeping hours (less than 6 h vs equal or more than 6 h)	0.270	< 0.001	0.170	1.252	
**Correlates of work-related burnout**					0.584
Age (> 40 years vs less than 40 years)	− 0.310	0.048	− 5.272	− 0.091	
Marital status (single/divorced vs married)	− 0.122	0.022	− 0.756	− 0.108	
Hospital type (private vs public)	− 0.067	0.001	− 1.277	− 0.037	
Gender (female vs male)	0.123	0.029	0.052	1.642	
Health condition (bad vs good)	0.297	0.018	0.139	1.121	
Working in the frontline (yes vs no)	0.379	< 0.001	0.101	0.582	
Diagnosed as COVID-19 case (yes vs no)	− 0.198	0.002	− 0.613	− 0.152	
Colleague diagnosed with COVID-19 (yes vs no)	0.325	0.008	0.187	0.457	
Threat perception scale	1.241	0.027	0.492	2.387	
Fear of COVID-19	1.055	< 0.001	0.842	1.568	
Altruistic (yes vs no)	− 0.418	0.023	− 0.753	− 0.215	
Low income	2.317	< 0.001	1.017	4.213	
Previous experience of working in outbreaks/pandemic	− 0.093	0.007	− 0.325	− 0.034	
Fear of COVID-19	1.993	0.002	0.916	3.018	
IFDFW scale	− 0.292	0.004	− 0.456	− 0.126	
Extensive working hours	1.671	0.027	0.814	3.543	
**Correlates of client-related burnout**					0.632
Age (> 40 years vs ≤ 40 years)	− 0.163	< 0.001	− 0.453	− 0.128	
year of experience (large vs small)	− 0.291	< 0.001	− 0.376	− 0.130	
Threat perception scale	1.953	< 0.001	1.543	2.712	
Altruistic (yes vs no)	− 0.267	< 0.001	− 1.312	− 0.106	
Low income	0.616	< 0.001	0.523	1.812	
Previous experience of working in outbreaks/pandemic	− 0.112	0.007	− 0.820	− 0.065	
Fear of COVID-19	1.431	0.018	1.054	2.617	
IFDFW scale	− 0.104	< 0.001	− 0.298	− 0.076	

Assumptions checked. Linear regression using the stepwise method. Variables included in the first step: age, gender, age, specialty, facility type, working in the frontline, presence of a child at home, presence of family member with chronic disease, income, health status, being diagnosed as a COVID-19 patient, previous experience of working in outbreaks, work experience, fear of COVID score, working hours, sleeping hours, Threat perception Scale, IFDWF wellbeing scale.

#### Interaction between TP of COVID-19 and financial wellbeing score

**Figure 3:**
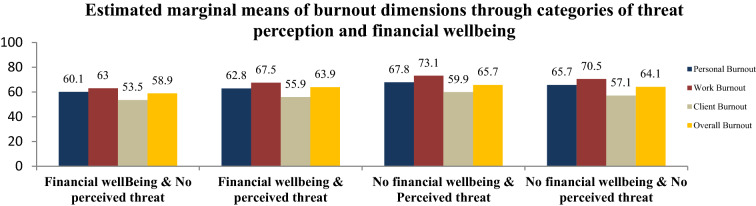
Estimated marginal means of burnout and its dimensions through categories of threat perception scale (low and high) and financial wellbeing (IFDFW).

The multivariate analysis showed a significant interaction between the TP of COVID-19 and the financial wellbeing (IFDFW) scores on estimated marginal means of burnout. Estimated marginal means showed a significant linear increase of contrasts between the four categories of physicians: those with no TP and IFDFW (Category 0), those with TP and IFDFW (Category 1), those with TP and no IFDFW (Category 2), and those with no TP and no IFDFW (Category 3). Compared to physician with no TP and financial well-being (CBI = 58.9; PB = 60.1, WB = 63, CB = 53.5), TP of COVID-19 added some burnout (CBI = 63.9, PB = 62.8, WB = 67.5, CB = 55.9) followed by a higher increase related to financial distress (CBI = 64.1, PB = 65.7, WB = 70.5, CB = 57.1) while the highest increase in burnout was found in subjects presenting high COVID-19 threat perception of and financial distress (CBI = 65.7, PB = 67.8, WB = 73.1, CB = 59.9) (Fig. 3).

## Discussion

The COVID-19 pandemic has aggravated the levels of burnout among physicians who had to shoulder the burden of COVID-19. The present study aims to assess the level of burnout among Lebanese physicians along with how sociodemographic, occupational, economic, and pandemic-related factors affect the intensity of burnout. Besides, it aimed to explore the combined effects of the pandemic and the economic crisis on burnout. It is believed that this paper is the pioneer study in Lebanon focusing on burnout during the context of double hit and investigating factors associated with burnout and the combined effect of these crises among physicians.

### Main findings

A significant burnout level was detected among physicians during these unprecedented times. A strong association was found between sociodemographic variables, occupational, economic, and exposure factors with higher levels of burnout. However, financial well-being, altruism, good health, and history of COVID-19 were significantly associated with lower levels of burnout. The analysis of the combined effect of the COVID-19 pandemic and financial wellbeing demonstrated that the presence of both TP and financial hardship significantly increased the level of burnout.

The findings of this study revealed that burnout hits more than 90% of the Lebanese physicians and around 20% suffered from a high level of burnout. Combining moderate and high levels of burnout, more than the third quarter of them expressed PB (mean = 64.8) and WB (mean = 71.5). As for CB (mean = 58.7), it was detected among 69.6% of participants. Several studies found in the literature documented burnout and its effects among physicians^72–75^ as well as its increasing trend of burnout during the pandemic. For example, a study reported that 45.8% of US physicians had experienced burnout^29^. Another study conducted among Austrian physicians showed a substantial increase of 30% in burnout rates during the pandemic compared to other studies conducted before the COVID-19 outbreak^7,8,45^.

In a systematic review covering 176 studies, an overall burnout rate of 48.7% was found^76^. Burnout syndrome was also found prevalent among 57.7% of Jordanian physicians^77^. Of note, the use of different tools for assessing burnout impedes the comparison of the results of this study directly with the findings of other previous studies such as the one conducted among Lebanese physicians in 2013^30^. Therefore, it was difficult to ascertain the increasing trend of burnout among the study population. In comparison with other studies using the CBI scale whether before or after the pandemic, the study’s findings were much higher than those reported in these studies^78,79^. For example, a study conducted among emergency physicians in Bahrein using the CBI scale found a prevalence rate of 81.0% for PB, 69.8% for WB, and 40.5% for CB^78^. Another study conducted among German general practitioners showed that one-third of physicians suffered from PB symptoms, one quarter showed WB while 12% of them reported a high prevalence of CBI^79^. Altogether, the crippling effect on mental health revealed by the alarming prevalence of burnout among Lebanese physicians was foreseeable. It could be understood in the light of the particular Lebanese context that cumulates the traumatic effect of the COVID-19^80^ and the unprecedented economic crisis. Hence, urgent measures that tackle this looming epidemic of burnout are required to save an already ailing health sector.

In terms of sociodemographic factors, our findings showed that higher burnout was associated with the female gender. However, studies in the literature reported dissimilar results in terms of gender. While a number of studies reported no gender differences in terms of burnout, other studies found that females experienced more burnout compared to males^2^ such as McMurray et al.^73^ who reported that women physicians had increased odds of burnout when compared to men. Consistently with the study findings, Kannampallil et al. also found a higher prevalence of burnout amongst women during the pandemic^81^. This could be explained by the high exposure to risk for female physicians given their predominance in patient-facing roles, gender expectations in care, with high workloads at their homes^82^.

Furthermore, this study highlighted the association between younger age and a high level of burnout. Our findings were consistent with the results of a study among Hungarian general practitioners and residents which considered younger age as the strongest predictor of burnout^83^. Conversely, another study conducted among Portuguese physicians reported that younger age and female gender were independent determinants of burnout^84^. Such a result could be explained by the fact that older physicians, learned during their journey, through their day-to-day practice and their previous encounters with stressful events how to anticipate, cope, and prepare for potentially tough situations. Therefore, it could be easier for them, than younger physicians to engage in their work, adopt positive adaptation, and apply emotion management skills^85^. To address this issue, specific programs to prevent burnout should be designed and implemented for physicians just starting their careers, such as coping and self-care strategies.

Another important aspect of burnout, noticed in this study was that being married decreased the level of burnout. The findings of Shanafelt et al.^29^ supported our results concerning the presence of a partner (being married) and the decreased risk of burnout^6^. This could be explained that physicians who are supported or feel supported by their partners or loved ones experienced less burnout when compared to those who do not. Interestingly another study showed that spouse support decreased burnout by 40%^3^. Further studies were suggested to explore the association between marital status and burnout.

Remarkably, having a dependent child or having a family member with comorbidities were both associated with higher burnout levels among physicians. Our results were comparable to those reported by Koh et al. and Maunder et al. both suggest that having children is a predisposing factor to burnout^4,5^. However, McMurray et al.^3^ found that women physicians who had young children to look after reported a decrease in burnout by 40%. The higher burnout level detected among these physicians could be explained by their concerns and anxiety about transmitting the disease to their vulnerable family members^86^.

In terms of pandemic-related factors, a higher TP was also associated with a higher level of burnout. This could be due to the uncertainty surrounding the pandemic in terms of healthcare policy reform and compensation changes that have the potential to instigate burnout. Overall, it is well recognized that intense fear and TP when people experience physical and psychosomatic disorders lead to anxiety, burnout, and emotional exhaustion^87–89^.

In terms of economic factors, a current low socioeconomic status and income, and negative financial well-being were found associated with a higher burnout level. Of note, a previous higher socioeconomic status and a current fear of poverty were found associated with higher stress and burnout, whereas current financial wellbeing was correlated with lower burnout^90–92^. Such penetrating association in low- and middle-income countries is leading to several mental disorders^93^. Of note, the Lebanese physicians with savings in the country’s banks and who were unable to reclaim their money represented a typical example. Moreover, the massive depreciation in the country’s currency led to a loss of more than 80% in physicians’ income^8^. It was revealed that the current situation had detrimental consequences among physicians, including soaring burnout, and psychiatric illnesses^94,95^ in addition to an exodus of physicians who left the country searching for stability, financial wellbeing, and safety. On other hand, the association between escalating poverty and economic insecurity and stress is well known^57^ which in turn, can lead to burnout and demission. Since the economic crisis is expected to escalate, health facilities were in danger of laying off employees, postponing some services, or completely closing their doors.

In terms of occupational factors, our findings showed that physicians who specialized in internal medicine and infectious diseases were more prone to suffer from higher levels of burnout compared to their colleagues. The role of specialties as a contributor to burnout found in this study may be partly due to differences in exposure to COVID-19 cases as ID specialists, and internal medicine physicians such as pulmonologists and cardiologists were more involved than other physicians in the treatment of COVID-19 cases. This dissimilarity of burnout among specialties was also highlighted by a meta-analysis conducted by Lee et al.^96^. Besides, the findings of this study highlighted that burnout rates were highest amongst physicians involved in frontline care. However, this was anticipated since their job presented a higher risk of infection due to their direct contact with COVID-19 cases. A study conducted by Kannampallil et al.^97^ showed similar results concerning the higher prevalence of burnout (46.3%) reported among physicians who were exposed to COVID-19 patients compared to those who were not exposed (33.7%)^14^. However, there are disparities regarding the correlation between burnout and working on the frontline^76^. For example, Wu et al.^28^ found that medical staff working on the frontline had a lower level of burnout compared to those working on usual wards explaining this unexpected trend, by suggesting that frontline workers may have felt a greater sense of control over the situation.

One peculiar finding in this study was that working in public hospitals was associated with higher burnout levels. This could be understood since public hospitals were firstly designated by health authorities to treat and isolate COVID-19 patients, hence physicians working in these hospitals were more exposed to COVID-19. In the light of the deep economic collapse which lead to a shortage of funds, the government was unable alone to support hospitals with much-needed resources and supplies. This called for the support of foreign and local non-governmental aid to import essential supplies and equipment, including personal protective equipment.

Similar to other studies, our findings showed that insufficient sleeping hours and extensive working hours were associated with a higher level of burnout^3^. In this regard, several studies highlighted that sleep deficiency is a key risk factor for burnout among physicians^18,98^. With the rise of COVID-19 cases, physicians are facing intense workload, and extensive working hours, which eventually impacted physicians sleeping hours. Of note, the role of sleep disorder was found, even in normal conditions, to be associated with four-fold bigger odds of burnout^17^.

In addition to the above, limited work experience was associated with a higher burnout level. Consistently, a Portuguese study showed that HCWs with larger experience were less affected by burnout^99^. Another study conducted among physicians in Lithuania found a significant reverse relationship between work and patient burnout and length of employment^100^. However, previous experience during a previous pandemic or emergency was associated with a decreased level of burnout among physicians. This could be explained that previous experience provides physicians with a sense of confidence and control over the situation and lessens their worries when dealing with patients. Physicians with good health status and previous history of COVID-19 experienced a lower level of burnout. Their good health status and a history of COVID-19 could lessen their concerns about their susceptibility as a previous infection could instigate their sense of being immune naturally.

The role of altruism in decreasing the level of burnout was supported by the study findings since physicians who accepted the risk of caring for COVID-19 cases had lower burnout levels in comparison with those who are not accepting this risk. Similar results were reported by a Turkish study that found a lower level of burnout among physicians who were actively involved in the fight against COVID-19 in comparison with their counterparts who are not actively involved^101^.

Lastly, the combined effect of the threat of the COVID-19 pandemic and financial hardship significantly increased burnout levels among physicians. Despite the scarcity of previous studies tackling such a topic, a review supported the effect of economic uncertainty on mental health in the era of COVID-19^64^. The increased risk of burnout among Lebanese physicians necessitates a combined approach to addressing the stressors resulting from the pandemic and economic crisis.

### Limitations

Several limitations should be acknowledged in our study. First, the study had a cross-sectional design which does not allow us to deduce causality. Selection bias is possible due to the snowball technique which limits the generalizability of the findings. The collected data was based on self-reported information which makes it prone to social desirability. Although taking into consideration of some potential confounders in the multivariable models, residual confounding is still possible. Face-to-face studies would be suggested in the future to confirm our results. Further longitudinal studies as well as following up on the burnout of Lebanese physicians would be recommended in the future to confirm our results, especially since the economic crisis escalates sharply in December 2020.

#### Implications for clinical practice and research

The alarming level of burnout detected among Lebanese physicians represented only the tip of the iceberg of the crisis in Lebanon. Its negative impacts that begin to effervesce with the exodus of some physicians would not be restricted to those healthcare providers but would also affect the patient’s quality of care and the healthcare organizations^20^. However, to date, there were no realistic evidence-based interventions and tangible measures that focused on physician burnout in Lebanon. The benefits of preventing physician burnout are not restricted to the affected individual and could also benefit the patient care as well as the overall health care system by potentially preventing physicians from leaving clinical practice. Hence, it is important to address factors identified by this study that potentially contribute to burnout among physicians in order to mitigate the long-term negative consequences through oriented strategies. However, these approaches counter Lebanese physician burnout and need to be further explored. It should empower the active involvement of the physician, at the facility level, in developing guidelines and designing contingency plans. These plans should create a supportive network and ensure physicians’ access to feedback channels as well as giving them to communicate with experts. Training on emotion management strategies should be performed to improve their preparedness for stressful situations. The importance of self-care (rest, healthy lifestyle, breaks, and sufficient sleep) should be recognized by the organization. The latter should screen regularly physicians at increased risk of personal and work-related burnout. In addition, government and health facilities should address this comorbidity among physicians through enacting proactive policies and providing critical leadership and funding for burnout prevention programs through a collaborative effort between national and institutional leadership. More studies exploring possible interventions based on physicians’ preferences and the feasibility of such interventions were recommended. The association between burnout and intention to leave clinical practice or to go abroad for clinical work would be recommended to be explored.

## Conclusion

After dealing with more than a year with the COVID-19 pandemic stressors combined with an unprecedented economic collapse, Lebanese physicians reached a crisis point and the problem is expected only to get worse in absence of urgent measures. This study found a huge and serious prevalence of burnout among Lebanese physicians which called for collaborative efforts from all stakeholders in healthcare to adopt urgent measures and to implement effective strategies to enhance the physicians’ wellbeing.

## Data Availability

After publication, the survey data will be made available on reasonable request to the corresponding author. A proposal with a detailed description of study objectives and a statistical analysis plan will be needed for the assessment of requests. Additional materials might also be required during the process of assessment.

## Abbreviations

COVID-19: Coronavirus disease 2019
CBI: Copenhagen Burnout Inventory
IFDFW: InCharge financial distress/financial well-being
FOC: Fear of COVID-19
HCWs: Health care workers
PPE: Personal protective equipment
PB: Personal burnout
WB: Work-related burnout
CB: Client-related burnout
MOPH: Ministry of Public Health
UK: United Kingdom
USA: United States of America
SPSS: Statistical package for social sciences
C.I: Confidence interval
SD: Standard deviation
α: Cronbach alpha
SARS: Severe acute respiratory syndrome
H1N1: Influenza A virus subtype H1N1
MERS: The Middle East respiratory syndrome
